# Perceived barriers to Chagas disease screening among a diverse group of prenatal care providers

**DOI:** 10.1371/journal.pone.0246783

**Published:** 2021-02-26

**Authors:** Helen Mahoney West, Carly E. Milliren, Olivera Vragovic, Julia R. Köhler, Christina Yarrington

**Affiliations:** 1 Division of Infectious Diseases, Boston Children’s Hospital, Boston, Massachusetts, United States of America; 2 Simmons University School of Nursing, Boston, Massachusetts, United States of America; 3 Institutional Centers for Clinical and Translational Research, Boston Children’s Hospital, Boston, Massachusetts, United States of America; 4 Boston University School of Medicine, Boston, Massachusetts, United States of America; 5 Department of Pediatrics, Harvard Medical School, Boston, Massachusetts, United States of America; 6 Department of Obstetrics/Gynecology, Boston Medical Center, Boston, Massachusetts, United States of America; Tulane University, UNITED STATES

## Abstract

**Background:**

Chagas disease is a vector borne infection of poverty endemic to Latin America which affects an estimated 40,000 women of child-bearing age in the United States (US). In the US Chagas disease is concentrated among individuals who have lived in endemic areas. Prenatal diagnosis and treatment are needed to prevent congenital transmission. The objective of this study was to assess perceived barriers to Chagas disease screening among prenatal care providers in Obstetrics/Gynecology and Family Medicine Departments of a tertiary care safety-net hospital caring for a significant at-risk population.

**Methodology/Principal findings:**

An anonymous survey was distributed to 178 Obstetrics/Gynecology and Family Medicine practitioners. Of the 66 respondents, 39% thought Chagas screening was very important, and 48% somewhat important as a public health initiative. One third judged screening patients during clinic visits as very important. Most respondents (64%) reported being familiar with Chagas disease. However, only 32% knew how to order a test and only 22% reported knowing what to do if a test was positive.

**Conclusions/Significance:**

These findings will be incorporated into measures to facilitate full implementation of Chagas screening, and can inform initiatives at other centers who wish to address this deeply neglected infection among their patient families. Greater integration of information on Chagas disease screening and treatment in medical and nursing education curricula can contribute to addressing this disease with the focus that its potentially fatal sequelae merit.

## Introduction

Chagas disease is a neglected tropical disease (NTD) caused by the parasite *Trypanosoma cruzi* (*T*. *cruzi*) which is transmitted to humans by triatomine insects. These vectors primarily live in thatched roofs, mud walls and cracks of homes made of natural materials in Latin America. For this reason, the disease is limited almost exclusively to low-income communities. Although infected mammalian reservoirs have been found in the Southern United States (US), the majority of infected individuals in the US have immigrated from Mexico, Central and South America [1]. In vector-free areas one of the main modes of transmission is mother-to-child, but transmission may also occur through blood transfusions, and bone marrow and organ transplantation [2,3]. An estimated 6–7 million cases of Chagas infection exist world-wide, mostly in Latin America [4–7]. The US has the sixth largest number of cases globally, estimated at over 300,000 [6]. Of these an estimated 40,000 are women of child-bearing age with an estimated 60–300 cases of congenital infection occurring annually [2]. The global healthcare costs of infected individuals are estimated to be $7–19 billion per year in 2012 dollars [8]. The US and Canada account for approximately 10% of Chagas related costs [8,9]. Lee et al. estimated the burden of both healthcare and work related absenteeism to be as high as $900 million in the United States [8].

Chagas disease has an acute and a chronic phrase. The acute phase is usually mild or asymptomatic but the parasite persists indefinitely. If untreated, an estimated 30% of infected individuals will progress to symptomatic heart disease, 10% to gastrointestinal disease, while less than 5% develop neurologic complications [10]. Symptoms may not appear for a decade or more. Individuals progressing to advanced cardiac disease, including cardiomyopathy, are at risk within 10–30 years of infection [9,11]. Importantly, diagnosing and treating women of childbearing age prevents congenital transmission, which occurs in 1–5% of pregnancies [12]. Although antiparasitic therapy with benznidazole has been shown to be effective in eradicating *T*. *cruzi* infection it is estimated that less than 1% of infected individuals have received treatment [13,14]. Recommendations to screen and treat women of child-bearing age and children with congenital disease [12,15–18] are rarely followed. The Centers for Disease Control and Prevention (CDC) website states “Women at risk for Chagas disease should be screened for infection before or during pregnancy. Women who have lived in Mexico, Central America, and South America are at greatest risk for Chagas disease” (CDC, 2020) [12].

There are many barriers to screening for and treating this disease. Non-adherence of medical providers to guidelines is but one of many barriers to screening and treating the disease. Chagas is a disease of poverty occurring primarily in a marginalized population of Latin American immigrants. Discrimination, dehumanization, disenfranchisement, fear of deportation, lack of insurance and a lack of investment in diseases of poverty in the US, contributes to the lack of attention to Chagas disease [4,19].

A paucity of provider awareness presents an additional barrier to diagnosing and treating Chagas disease [20]. A survey of US physicians including primary care, infectious diseases, cardiology, obstetrics-gynecology and transplant practitioners found a general lack of knowledge of Chagas disease among all groups. However, lack of awareness of this disease was most pronounced (47%) among obstetricians surveyed [20]. In another study assessing knowledge of the disease among Obstetrics/Gynecology (Ob/Gyn) providers, the researchers found that 68% of respondents reported “very limited” knowledge of Chagas disease, with only 9% reporting awareness of the risk of congenital Chagas infection [21]. Additionally, a survey of US Pediatric Infectious Diseases Society members reported that respondents “seldom consider the risk of congenital Chagas disease in infants of parents from Latin America” (Edwards et al., 2018, p. e26) [22]. These studies underscore the need for increased Chagas disease education among healthcare providers as a means of improving the identification, screening and treatment of at-risk populations [21].

The multistep process by which Chagas disease is diagnosed serologically is another barrier to care. Screening Enzyme-Linked Immunosorbent Assays (ELISAs) are available in commercial laboratories, but because of their low specificity, a positive screening result requires confirmatory testing. For confirmatory testing, which is conducted at the CDC, a specific CDC sample submission form must be filled out and sent, together with the serum sample, to a State Public Health laboratory, which then ships the sample to the CDC. Results from the CDC return by fax, so these results must be monitored and entered into patients’ electronic medical records. Providers intending to screen their obstetric patients for Chagas disease must master this multistep process, and they must know where in their institution’s electronic medical record system the cognate orders, the form and the results are accessible.

Considering the morbidity, mortality and costs associated with the disease, improving rates of Chagas disease screening and treatment has the potential to prevent congenital transmission, improve health outcomes and reduce costs associated with caring for individuals infected with the parasite. The aim of this study was to identify barriers to Chagas screening among Ob/Gyn, and Family Medicine practitioners. It is hoped that this study will raise awareness of the disease and inform initiatives for improving rates of Chagas disease screening in at-risk populations, especially women of child-bearing age.

## Methods

This study was approved with exempt status by the Institutional Review Boards at Boston Children’s Hospital (IRB Protocol # IRB-P00032813) and Boston Medical Center (Protocol # H-39472). Study participants were informed that their participation in the survey was anonymous and voluntary and consent was inferred by their participation.

### Setting

The study was conducted at Boston Medical Center (BMC) through the Chagas disease “Strong Hearts” project. “Strong Hearts” is a joint project between Boston Children’s Hospital (BCH), East Boston Neighborhood Health Center and BMC. To our knowledge, to date this is the only program in the United States that has conducted Chagas educational sessions followed by implementation of Chagas screening and referral for treatment in both a Primary Care and Ob/Gyn clinical setting.

Boston Medical Center is a non-profit academic medical center in Boston, Massachusetts and is the largest safety-net hospital in New England. Chagas disease screening is based on each individual clinician’s initiative and familiarity with the disease. Once a patient is diagnosed with Chagas disease through confirmatory testing at the CDC, they are referred to an Infectious Diseases specialist at BMC for evaluation and, if appropriate, antiparasitic therapy with benznidazole or nifurtimox.

### Sample

The convenience sample included Ob/Gyn and Family Medicine physicians (including residents), midwives, Ob/Gyn nurse practitioners (NPs), and Ob/Gyn ambulatory registered nurses (RNs).

### Design

A survey was designed to assess provider familiarity with Chagas disease and perceived barriers to Chagas screening. Familiarity with who is at risk, how to screen and what to do if a patient tests positive were of primary interest. The survey followed educational sessions for Ob/Gyn providers about Chagas disease and its congenital transmission that began more than 2 years earlier. An invitation to participate in an anonymous electronic survey was sent via email by the director of Labor and Delivery. This was a closed survey of a targeted group of providers at one institution. Ob/Gyn and Family Medicine providers who provide prenatal care to women at risk for Chagas disease were the target population. The survey was voluntary and completely anonymous with no identifying information collected. No incentives were offered and the survey was designed to take approximately 5–10 minutes to complete. Consent to participate was indicated by the participant’s willingness to complete and submit the survey. The survey was open for eight weeks, February and March 2020 and weekly reminders were sent via email to potential participants. Additionally, reminders of the opportunity to participate in the survey was provided by word of mouth by the Labor and Delivery director to potential participants. Participants were able to skip questions and to stop taking the survey at any time. This study was considered exempt by the institutional review boards (IRB).

The survey included a total of 17 items. Categories included demographics of respondent, familiarity with Chagas disease, importance of Chagas screening as a public health initiative, perceived barriers to testing during clinic sessions, who should contact patient with test results, who should coordinate referral for treatment if test is confirmed positive, and recommendations for improving Chagas disease screening (Appendix A.) Survey items demonstrated high internal consistency within specific domains (Cronbach alpha = 0.82 and 0.85 for familiarity and barrier items respectively) and overall across items (Cronbach alpha = 0.79 for all familiarity, barrier and importance items).

Study data was collected and managed electronically using Research Electronic Data Capture (REDCap) hosted at BCH. REDCap is a secure, web-based software platform designed to support data capture for research studies and electronic surveys.

### Statistical analysis

Frequency and percent are reported for all categorical survey items. Agreement between familiarity items were tested using McNemar chi-square test. Associations between familiarity items and barrier items were examined using the Cochran-Armitage test of trend to test for differential familiarity across ordinal Likert-scaled barrier responses. Similarly, the association between overall public health importance and familiarity items was assessed using the Cochran-Armitage test of trend to test for differential agreement across ordinal Likert-scaled importance categories. The association between overall public health importance and barriers was assessed using the Mantel-Hanszel chi-square test for trend as both row and column were ordinal Likert-scaled categories. All analyses were performed in SAS (version 9.4; Cary, NC).

The survey instrument, final dataset and analysis code are stored in a repository on Open Science Framework and available at the following link: https://osf.io/fqg8n/?view_only=1c4d8c1beb6e4fe39e67a7f5961e04f1

## Results

**Fig 1:**
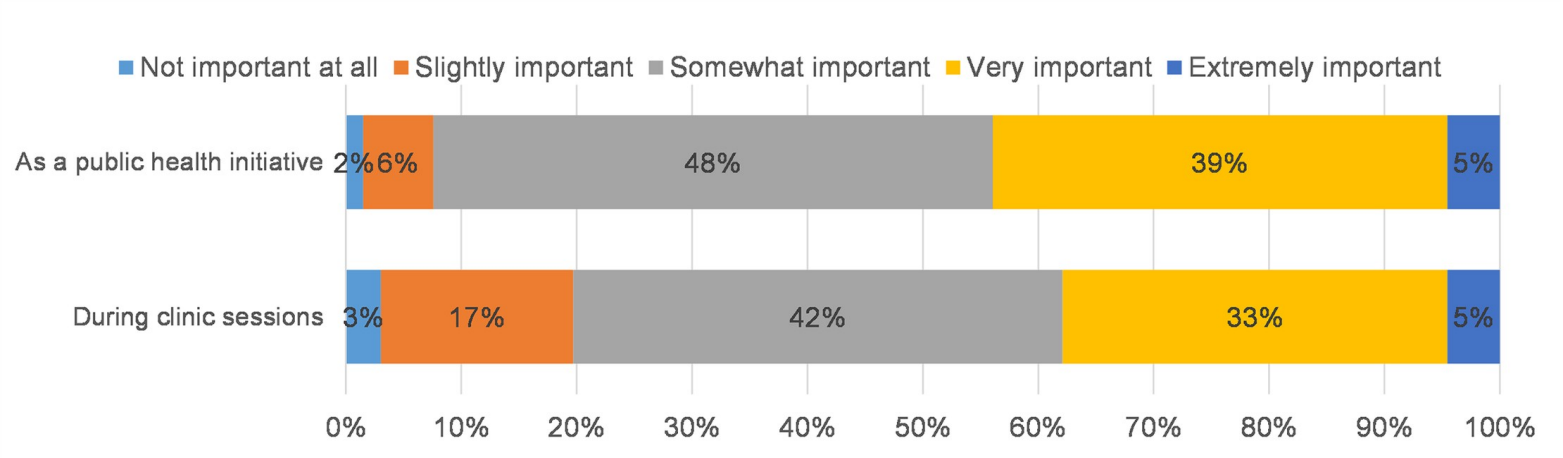
Importance of Chagas screening (N = 66).

**Fig 2:**
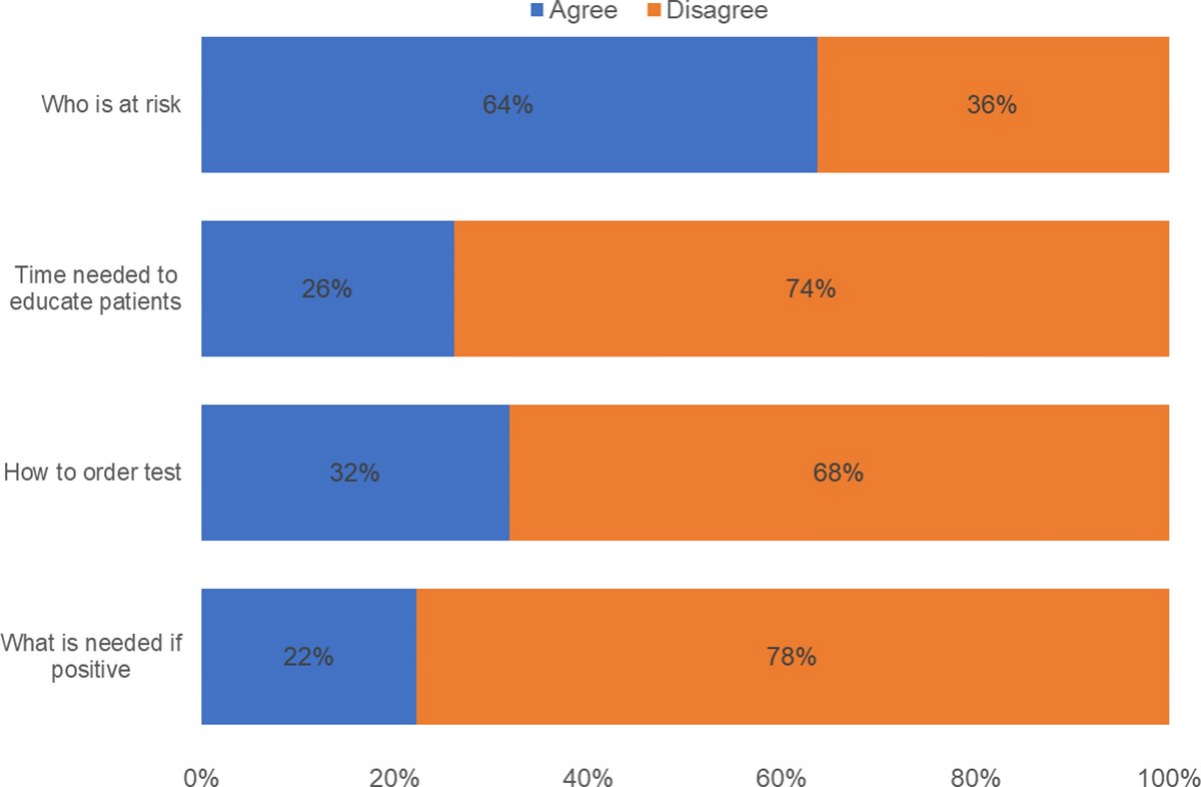
Familiarity with Chagas screening (N = 66).

The survey was sent to a total of 178 Ob/Gyn and Family Medicine providers and 66 (37%) completed the survey. The respondents were made up of 44 physicians (67%), 12 midwives (18%), 4 NPs (6%) and 6 unknown provider type (9%). Almost 90% of participants supported screening: 39% responded that Chagas screening is very important, and 48% that it is somewhat important as a public health initiative. Three quarters endorsed the importance of screening at the time of clinic visits with 33% identifying screening as very important and 42% somewhat important. One respondent thought that it was not important at all to screen and 2 thought that it was not important to screen during clinic sessions (Fig 1). Of the 66 respondents, 64% reported being familiar with who is at risk for Chagas disease. However, only 32% knew how to order a test; 22% knew what to do if a test was positive and only 26% were familiar with the time needed to educate patients (Fig 2).

**Fig 3:**
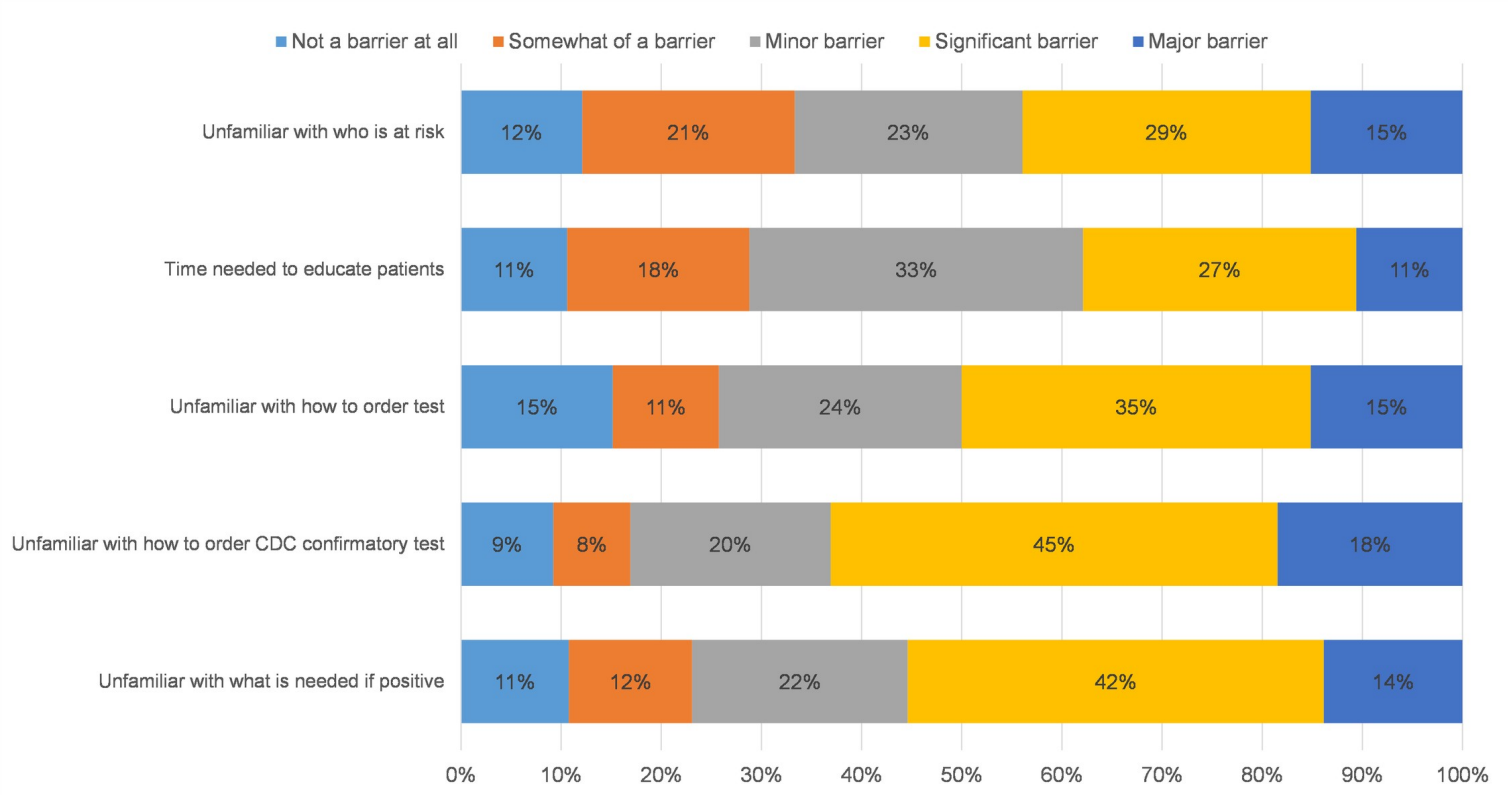
Barriers to Chagas screening (N = 66).

Being unfamiliar with patient populations at risk was identified as either a major (15%) or significant (29%) barrier to testing. Over half of the respondents identified time needed to educate patients as either a major (11%), significant (27%) or minor barrier (33%). Forty percent (40%) of respondents reported being unfamiliar with how to order a test as either a major (15%) or significant (35%) barrier. Lack of knowledge concerning how to order a CDC confirmatory test was substantial for over half of respondents, being noted as a major (18%) or significant (45%) barrier. Likewise over half of respondents reported being unfamiliar with what to do if a test returned positive as a major (14%), significant (42%) or minor (22%) barrier to testing (Fig 3).

**Fig 4:**
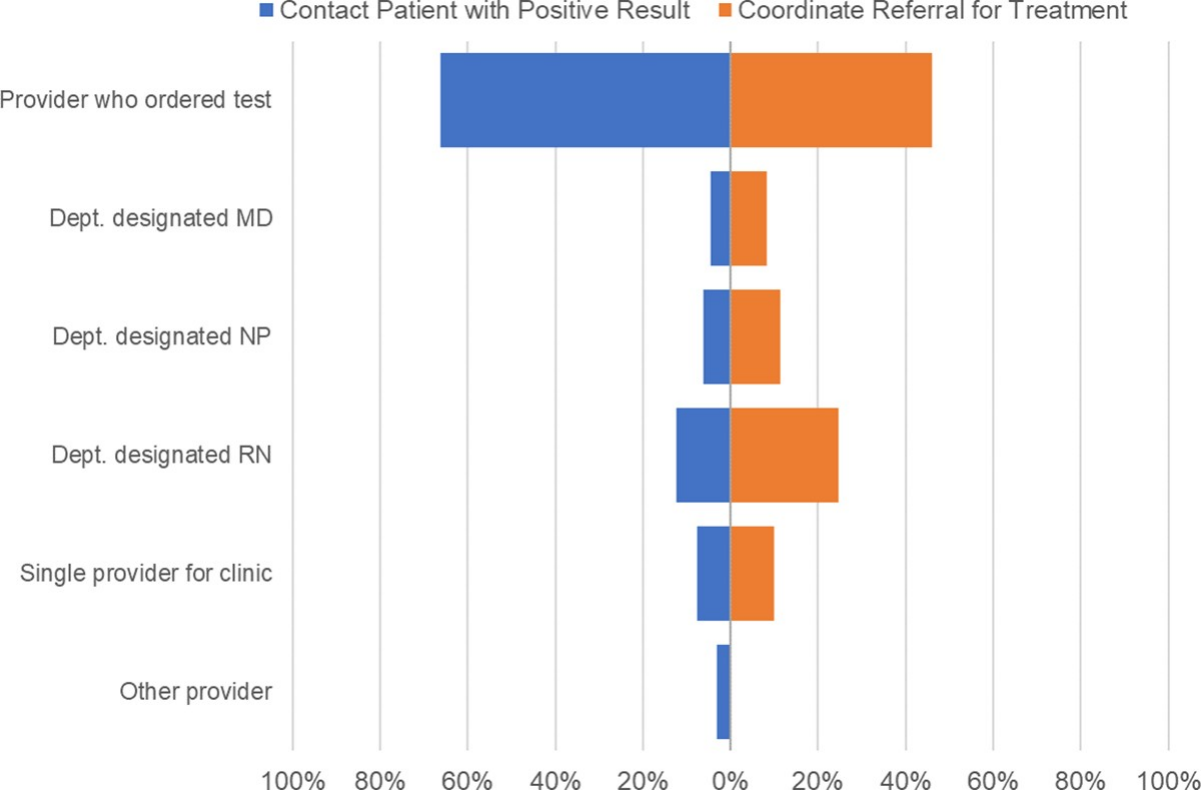
Provider perceptions of who should contact patient with positive result and coordinate treatment (N = 66).

Respondents overwhelmingly (90%) felt that the provider seeing a patient should order screening tests. The majority of respondents (66%) thought that the provider who ordered a Chagas test should contact patients with results and 46% replied that the provider who ordered the test should coordinate referral for treatment (Fig 4).

Only 29% of respondents knew both who was at risk and how to order the test: 26% knew both who is at risk and time needed to educate the patient, and 22% knew both who is at risk and what to do if the test is positive. Lack of knowledge was perceived as a barrier to care, since providers who were not familiar with who is at risk were more likely to identify it as a barrier (p = 0.003). Providers unfamiliar with how to order a test were more likely to identify the ordering process as a barrier (p<0.001), while those unfamiliar with what to do if a test result was positive were more likely to identify lack of knowledge about what to do with a positive result as a barrier (p<0.001).

Providers endorsing familiarity with who is at risk and the time needed to educate patients were more likely to rate screening as more important (p = 0.001, and p = 0.004 respectively). There was no difference in rating of importance by familiarity with next steps when a test is positive (p = 0.32). There were no differences by provider type for any of the familiarity or barrier items. No association between any of the barrier questions and the overall public health importance rating was found. Familiarity with time needed to educate was not associated with respondents’ perception of who should screen.

## Discussion

The purpose of this study was to identify perceived barriers to screening for Chagas disease among prenatal care providers via an anonymous electronic survey. Ob/Gyn and Family Medicine providers are front line clinicians who care for patients vulnerable to Chagas disease and are positioned to increase screening in pregnant women, women of child-bearing age, their infants, older children and families. By implementing screening, they can implement a life-saving intervention for their patients, prevent mother to child transmission and identify infants at risk for congenital infection in order to diagnose, treat and cure them [23].

Results from this survey indicated no difference by provider type and that an overwhelming majority of these clinicians felt that Chagas disease screening is very or somewhat important. Yet many affirmed lacking the tools to act on this goal. Nearly two-thirds of respondents were familiar with Chagas disease, while only one-third knew how to order a test and less than one-third knew what to do if a test was positive. Of those who reported knowing who is at risk, two-thirds did not know what to do if the screening test was positive and over half did not know how to order the test or the time needed to educate patients. Survey participants recognized that their lack of knowledge presented a barrier to screening their patients for this disease; being unfamiliar with who is at risk, time needed to educate patients, how to order an initial and follow up CDC confirmatory test, and next steps when a test is positive were all identified as barriers to Chagas disease screening. Being unfamiliar with patients at risk was also noted by Stimpert and Montgomery, Verani et al., and Edwards et al. [20–22].

Additionally, the majority of respondents indicated that the provider seeing a patient should order screening tests and contact patients with results. This is the practice standard at BMC. However, unless a provider is familiar with a disease, divulging results may be overwhelming, as was the case with the Zika epidemic. Anecdotally, many providers have expressed a desire to have an individual with Chagas expertise available to field patient questions. To address this issue, a script has been built into the electronic medical record to assist providers when discussing results with patients. When considering barriers to Chagas screening and treatment the potential for losing patients to follow-up must also be considered and appropriate steps taken to prevent this outcome.

This study follows previous research indicating a lack of awareness of Chagas disease among providers as a barrier to care [20–22]. Previous studies have identified a lack of awareness of Chagas disease among obstetrician-gynecologists including pathophysiology, epidemiology, and populations at-risk [20,21]. Our findings suggest there is an additional dimension that has been missing from provider education, namely the complex steps needed to make a Chagas diagnosis. This requires training in logistics, including how to order a Chagas test and how to follow-up on positive test results. Many of the highly motivated participants in our study, with a mission of serving the underserved, had not yet attained this expertise.

This study has a number of limitations. The small, targeted provider sample with a response rate of only 37% limits the ability to generalize the results of this study to the wider provider population. Although several educational events were provided at BMC over the course of a couple of years, and clinicians in Ob/Gyn and Family Medicine expressed significant awareness of the disease and embraced screening following these sessions, we do not know how many of the survey respondents had participated in these sessions. Hence exposure to these educational sessions is a potential confounding factor that we did not assess. Potentially, the clinicians who already had an interest in Chagas disease were over-represented among the respondents. Further, although MDs were overrepresented in the sample we found no major differences in responses to questions associated with familiarity or barriers between MDs and other providers. Data on respondents’ department, Ob/Gyn or Family Medicine was not collected. Knowledge of participants’ medical specialty could provide additional insight into whether a difference in familiarity with Chagas disease exists between departments. Additionally, we do not know the department of the providers who did or who did not complete the survey and therefore it is unknown if the results are biased toward one discipline. Emails were not captured, potentially allowing for respondents to take the survey multiple times. However, although the public survey link allowed for respondents to complete the survey more than once, the survey was only open for six weeks reducing the potential for repeat respondents; this also seems unlikely given clinicians’ time constraints.

To overcome barriers identified by the respondents to our survey, continuous education in the pragmatic details of the diagnostic process should be undertaken. Integrating information about Chagas disease into professional seminars, continuing education initiatives, medical and nursing school curricula, are further strategies to increase awareness of this disease among clinicians. In addition, professional and institutional support is needed to incorporate Chagas disease screening into patient visits, and to facilitate laboratory testing for the disease.

More fundamentally, to support implementation of one-step testing, similar to the testing process for infections like Lyme disease and HIV, Chagas disease should be recognized as prevalent in many regions of the United States and therefore deserves to be acknowledged as important within the medical system. Once testing is implemented more broadly, higher-specificity screening tests will be demanded and deployed by clinicians intending to provide comprehensive and life-saving care to their patients of Latin American origin. Importantly, in addition to addressing barriers within the medical system, studies that identify and address barriers to patients (e.g. access, insurance, immigration status) that contribute to delays and gaps in treatment or in loss of patients to follow up are needed.
